# The clinical value of CT and MRI in preoperative TNM staging of esophageal cancer

**DOI:** 10.3389/fmed.2025.1637764

**Published:** 2025-09-09

**Authors:** Weijun Zhao, Jing Huang, Yukun Chen, Jingjing Lu, Wentao Hu

**Affiliations:** ^1^Department of Thoracic Surgery, The First Affiliated Hospital of Ningbo University, Ningbo, China; ^2^Department of General Surgery, Shanghai Changhai Hospital, Shanghai, China; ^3^Department of Radiology, Shanghai Changhai Hospital, Shanghai, China; ^4^Department of Radiotherapy and Oncology, Affiliated Kunshan Hospital of Jiangsu University, Kunshan, China

**Keywords:** esophageal cancer, CT, MR, diagnosis, radiology

## Abstract

**Objective:**

To evaluate and compare the clinical value of computed tomography (CT) and magnetic resonance imaging (MRI) in the preoperative TNM staging of esophageal carcinoma.

**Methods:**

A total of 1,209 patients with histologically confirmed esophageal cancer who underwent preoperative CT (*n* = 868) or MRI (*n* = 341) scanning from January 2014 to December 2024 were retrospectively analyzed. Tumor location, histologic type, and postoperative pathological TNM stage were recorded. CT and MRI findings were compared with pathological results as the standard.

**Results:**

For T-stage, the overall accuracy of CT was 86%, with a sensitivity and specificity of 53 and 89%, respectively. MRI achieved an accuracy of 82%, with sensitivity and specificity of 57 and 93%, respectively. For N-stage, CT showed a sensitivity, specificity, and accuracy of 85, 83, and 84% for mediastinal lymph nodes, and 87, 82, and 83% for abdominal lymph nodes. There was no significant difference in the total accuracy rate of TN staging diagnosis of esophageal cancer between the two groups.

**Conclusion:**

Both CT and MRI demonstrate high diagnostic value in the preoperative TNM stage of esophageal carcinoma. While CT is more economic and easier to operate, MRI offers superior contrast of connective tissues and multiplanar imaging, making it particularly valuable in evaluating T4 stage and assessing tumor invasion of adjacent organs. Limitations exist in early T-stage and N-stage assessments due to difficulty distinguishing mucosal infiltration and differentiating metastatic from non-metastatic lymphadenopathy. Multimodal imaging strategies and tumor-targeted contrast agents may enhance staging precision.

## Introduction

Early-stage esophageal cancer patients usually show no obvious symptoms, by the time they are diagnosed, most are already in the late stage, leading to a large variation in prognosis (1). Early-stage esophageal cancer, if treated with radical resection, is expected to achieve long-term survival. In contrast, for some advanced cases, surgery may be futile or even harmful, increasing patient trauma and hastening death (2). Therefore, how to diagnose esophageal cancer early and accurately stage it preoperatively has become a major and difficult task in the field of esophageal surgery. Accurate preoperative staging helps surgeons select appropriate treatment strategies (3).

To allow early-stage esophageal cancer patients to undergo radical surgical treatment, and to avoid unnecessary or palliative surgery and chemoradiotherapy in late-stage patients, many imaging methods have been applied in clinical diagnosis of esophageal cancer, such as CT (computed tomography), MRI (magnetic resonance imaging), EUS (endoscopic ultrasonography), PET (positron emission tomography), thoracoscopy, and laparoscopy (4). Researchers have been conducting studies on the accuracy of these methods in TNM staging of esophageal cancer, but there is still no consensus. Among these, CT and MRI are the most widely used (5). This study compares preoperative CT and MRI findings with intraoperative and postoperative pathological results, identifying their respective advantages and limitations, in order to guide clinical practice.

## Methods

### Clinical data

This is a retrospective study included patients from January 2014 to December 2024, a total of 1,209 patients with esophageal cancer underwent preoperative TNM staging via CT (868 cases) or MRI (341 cases). All patients underwent postoperative gross pathology examination for TNM staging, which was compared with the preoperative CT and MRI results. Two radiologists jointly examined the CT and MRI images. When there was a dispute, a third senior radiologist would re-examine the images.

### CT scanning

CT examinations were performed using a spiral CT scanner (Hispeed CT/i, GE, USA), with a slice thickness and pitch of 5 mm. The scanning range extended from the supraclavicular apices to the upper pole of the adrenal glands, including the entire liver. Contrast-enhanced scans were performed in dual phases, with intravenous injection of 50 mL of iohexol. For lower esophageal lesions, 300 mL of deionized water was administered orally before the scan to distend the stomach.

### MRI scanning

MRI was conducted by a 1.5 T superconducting scanner (Gyroscan 5NT, Philips, Netherlands). Multisection spin echo sequences were obtained with TRs of 500, 1800, or 2,500 ms and TEs of 35, 90, or 120 ms. T1-weighted images (T1W) used TR 500 ms and TE 30 ms, while T2-weighted images (T2W) used TR 1500–1800 ms and TE 90–120 ms. Slice thickness was 4–6 mm, with a 1,024 × 1,024 matrix. Coronal, sagittal, and axial views were routinely obtained, with the scan range identical to CT. To ensure gastric distention, patients ingested 0.59 g/kg of Gd-DTPA before scanning.

### CT and MRI staging criteria

**Figure 1:**
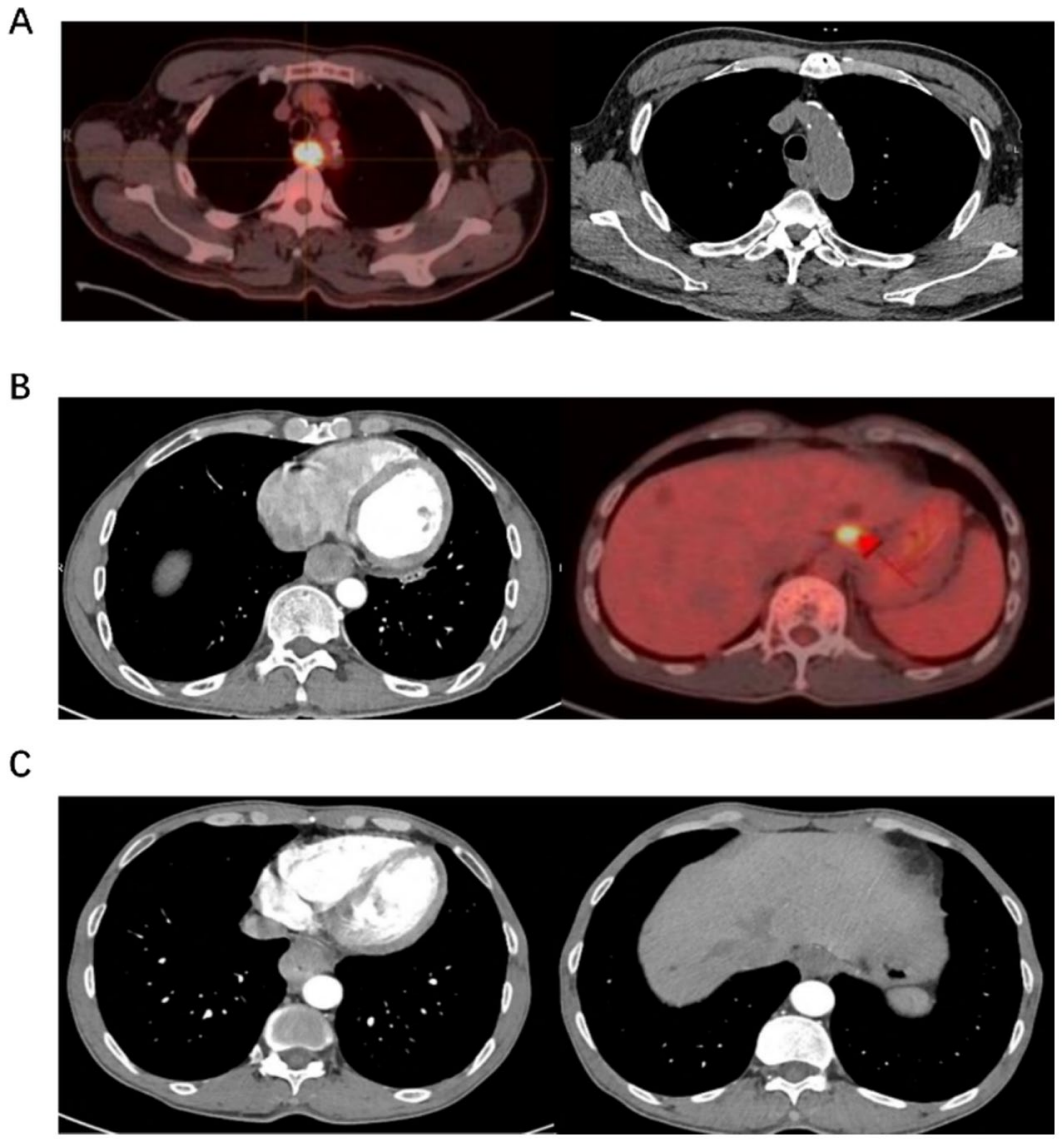
Examples of diagnostic diagram. **(A)** It shows a PET/CT scan with a bright area indicating activity in the chest, alongside a CT scan of the chest. **(B)** It displays a CT scan highlighting internal organs and a PET scan with a bright spot in the liver area. **(C)** It presents two CT scans, one showing the heart and another showing diferent sections of the torso.

The T staging criteria were based on the method of AJCC TNM staging. Tumor thickness of 3–5 mm was classified as T1, 5–15 mm as T2, and >15 mm with irregular outer esophageal margins as T3 (6). Involvement of adjacent organs such as the trachea, aorta, or vertebrae was defined as T4. Lymph nodes with short-axis diameters >10 mm were considered abnormal. Figure 1 shows images without lymph node metastasis, cardiac lymph node metastasis, and left gastric lymph node metastasis (Figure 1).

### Statistical analysis

Postoperative gross pathology results were used as the final standard to calculate the sensitivity, specificity, and accuracy of CT and MRI. Statistical significance was assessed with the *t* test and chi-square test, with *p* < 0.05 considered significant.

## Results

From January 2014 to December 2024, a total of 1,209 patients with esophageal cancer underwent preoperative TNM staging via CT (868 cases) or MRI (341 cases). There were no differences in gender, age, BMI, tumor height and pathological types between the two groups (Table 1).

**Table 1:** Clinical and pathological parameters of both groups.

Parameter	CT *n* = 868	MRI *n* = 341	*p* value
Age	57.98 ± 12.66	62.95 ± 11.50	0.052
Gender			
Male	554 (63.82%)	209 (61.29%)	0.202
Female	314 (36.18%)	132 (38.71%)	
BMI	23.38 ± 3.24	23.54 ± 2.81	0.798
Tumor height			
Upper	99 (11.41%)	36 (10.56%)	0.848
Middle	470 (54.15%)	197 (57.77%)	
Lower	299 (34.44%)	108 (31.67%)	
Pathological type			
Squamous carcinoma	802 (92.40%)	311 (91.20%)	0.052
Adenocarcinoma	56 (6.45%)	6 (1.80%)	
Sarcomas	3 (0.35%)	1 (6.12)	
Adenosquamos	7 (0.8%)	3 (0.88%)	

### Comparison of CT and MRI accuracy in T staging

Among 868 patients who underwent CT, the distribution of preoperative T stages was T1 (57), T2 (114), T3 (340), and T4 (357). Of 57 patients with pathological T1 disease, 14 were overstaged, 13 were not identified, 30 was accurately diagnosed as T1 (53%), and 72 as T2 (63%). Among 340 pathological T3 cases, 12 were understaged as T2, 2 as T1, 13 as T4, and 303 correctly staged (89%). Of the 357 T4 cases, 26 were misdiagnosed, yielding a correctly staging rate of 93%. The overall accuracy of 86% (Table 2).

**Table 2:** Comparison of clinical T stage and pathological diagnosis.

T Stage	CT		MRI		*p* value
	Patients	Accuracy	Patients	Accuracy	
T1 (*n* = 89)	57	53%	32	53%	0.819
T2 (*n* = 167)	114	63%	53	66%	0.675
T3 (*n*-501)	340	89%	161	86%	0.592
T4 (*n* = 452)	357	93%	95	98%	0.231
Summation	868		341		

For MRI (341 patients), T1 (32), T2 (53), T3 (161), T4 (95). Among 32 pathological T1 cases, 7 was missed, and 8 overstaged, with a correct diagnosis rate of 53%. For 53 T2 cases, 8 was understaged, 9 overstaged, 36 correctly staged (66%). Among 161 T3 cases, 11 was understaged, 12 overstaged, and 138 correctly staged (83%). 93 T4 cases were accurately staged. MRI overall accuracy of 82% (Table 2).

### Comparison of CT and MRI accuracy in N staging

Among the patients who underwent CT examination before the operation, 597 cases were pathologically confirmed mediastinal metastasis. 383 cases (84%) were diagnosed by CT. Among the 612 cases without lymph node metastasis, 363 cases were correctly diagnosed by CT (88%). There were 346 cases of abdominal lymph node metastasis, and 255 cases (90%) were diagnosed correctly by CT. The accuracy of CT in diagnosing abdominal lymph node metastasis was 90%.

Among the 341 patients who underwent MRI examination before the operation, 141 cases of mediastinal lymph node metastasis and 96 cases of abdominal lymph node metastasis were confirmed by pathology. The accuracy of MRI in diagnosing lymph node metastasis is in the mediastinum and abdomen are 84 and 86%, respectively (Table 3).

**Table 3:** Comparison of clinical N stage and pathological diagnosis.

Pathological	CT		MRI		*p* value
	Patients	Accuracy	Patients	Accuracy	
Mediastinal					
No (*n* = 612)	363/412	88%	170/200	85%	0.675
Yes (*n* = 597)	383/456	84%	118/141	84%	0.892
Abdominal					
No (*n* = 863)	507/618	82%	196/245	80%	0.723
Yes (*n* = 346)	225/250	90%	83/96	86%	0.652

## Discussion

After the diagnosis of esophageal cancer, it is very important for clinical surgeons to accurately determine the TNM stage of the tumor to choose the individualized treatment plan (7). In recent years, with the popularity of CT and MRI, it has brought many benefits for esophageal surgeons and patients to choose the appropriate treatment mode, but its accuracy in preoperative TN staging of esophageal cancer has been controversial (8).

It can be seen from the results of this study that there is no difference between CT and MRI in preoperative TN staging of esophageal cancer (with postoperative gross pathological results as the gold standard) in terms of sensitivity, specificity and accuracy (x test, *p* > 0.05). The advantage of CT is that the imaging technology is simple, the parameters are less, and it is easy to be grasped by clinicians. The price is relatively low. Enhanced CT scan can also show the relationship between the mass and the large blood vessels of the heart, making up for the lack of traditional X-ray examination, and the vast majority of cases can be diagnosed with X-ray and CT results. However, for some special cases, conventional CT scan has limitations, so MRI examination is necessary. MRI has the advantages of being multi-directional (axial, sagittal, coronal or even oblique), showing tumor components, the relationship between tumor and blood vessels, and the blood vessels within the tumor (9). The disadvantages are that MRI has many imaging parameters, is susceptible to interference by external factors (breathing, heartbeat), and is expensive (10).

A large number of literatures have reported that the accuracy of CT and MRI for preoperative T staging of esophageal cancer ranges from 45 to 73% (11). The results of this study showed that for T1 and T2 tumors, the diagnostic coincidence rate of CT and MRI was not high, especially when T1 tumors were confined to the esophageal mucosa or submucosa, (12) the tumors may only cause changes in the local motor function of the esophagus without changes in the thickness of the esophageal wall, while CT and MRI could only perform static imaging of the esophagus, but could not perform dynamic observation. Therefore, there is a high error rate (13). The low accuracy for T1/T2 staging may due to the atypical imaging features of early-stage lesions. Quint et al. (14) proposed that the normal wall thickness of the esophagus was 3 mm, the anteroposterior diameter was 14 mm, and the left and right diameter was 18 mm when the esophagus collapsed. At present, most domestic and foreign scholars still determine the depth of tumor invasion by observing the thickness of the esophageal wall according to the standard, which is obviously unable to keep up with the development of modern science and technology. How to improve the diagnostic technology of CT and MRI, determine more accurate diagnostic criteria of CT and MRI for esophageal cancer, and achieve early detection of esophageal cancer and screen the depth of tumor invasion still needs to be further discussed by radiologists. Of course, this is one of the main reasons for the current limitations on CT and MRI as alternatives to esophageal barium meal and endoscopy. EUS examination is recommended for patients with suspected early esophageal cancer to improve the accuracy of preoperative staging (15). In the future, we will further combine the EUS with MRI to improve the accuracy.

Although neither CT nor MRI can accurately distinguish each layer of the esophagus and judge the depth of tumor invasion, when esophageal cancer develops to the T3 and T4 stages, CT and MRI can not only show the subtle difference in the density of the tumor and its surrounding tissues, but also display the tumor in multiple directions and carefully distinguish its degree of external invasion in spatial orientation (16). In particular, the accuracy of T4 esophageal cancer was especially high (the T coincidence rate of CT and MRI diagnosis in this group was as high as 92 and 100%, respectively). In addition, CT and MRI can accurately determine whether esophageal cancer has invaded adjacent organs (trachea, bronchus, aorta, pericardium, etc.) and the extent of invasion. Since results demonstrated similar accuracy of CT and MRI, we recommend CT scan for initial screening for its economical and quick characteristic, and MRI for suspected T4 or complex anatomical sites, such as aortic invasion. If cases of suspected T4 stage located at regions with limited medical resources, CT can be the alternative for diagnosis.

CT and MRI have incomparable advantages over traditional X-ray in N staging of esophageal cancer. It is generally believed that pathological enlargement of thoracic lymph nodes >1 cm, subclavian lymph nodes > 0.6 cm, and abdominal lymph nodes > 0.8 cm are pathological enlargement. Due to the high resolution of CT and MRI, the diagnosis rate of mediastinal and abdominal enlarged lymph nodes is relatively high (specificity, sensitivity and diagnostic coincidence rate are >80%), which can provide clinical reference. However, CT and MRI still have high false negative and false positive results. In clinical practice, enlarged lymph nodes are not metastatic lymph nodes, while metastatic lymph nodes are not enlarged. Previous literature reports also confirmed the existence of high false negative and false positive rates in CT and MRI. Therefore, there is still a serious shortage in judging the N stage of esophageal cancer from the size of lymph node findings on CT and MRI alone. The development of tumorophilic contrast imaging is expected to further improve the accuracy of CT and MRI in preoperative TNM staging of esophageal cancer. However, we did not compare the equipment heterogeneity across hospitals. In the future, we will promote the results of this research to other medical centers and conduct a multi-centered prospective study.

## Data Availability

The raw data supporting the conclusions of this article will be made available by the authors, without undue reservation.
